# Time to Listen: Most Regular Patrons of Music Venues Prefer Lower Volumes

**DOI:** 10.3389/fpsyg.2019.00607

**Published:** 2019-03-22

**Authors:** Elizabeth Francis Beach, Megan Gilliver

**Affiliations:** ^1^National Acoustic Laboratories, Macquarie University, Sydney, NSW, Australia; ^2^The HEARing Cooperative Research Centre, Melbourne, VIC, Australia

**Keywords:** live music, nightclubs, sound levels, loudness preferences, hearing loss, tinnitus, leisure noise, music venue patrons

## Abstract

High sound levels are a feature of nightclubs and live music venues, and therefore pose a risk to patrons’ hearing. As a result, these venues are often a focus area for hearing health promotion, and particular emphasis is placed on motivating patrons to take steps to reduce their noise exposure. In the current study, we approached this issue from a different angle. We asked whether sound levels in music venues accurately reflect the preferences of regular patrons, and examined their attitudes and preferences toward sound levels and protective listening behaviors. The study examined results from 993 regular patrons of nightclubs and live music venues, collected as part of an Australian online hearing health survey. Participants were asked about their participation at the two target venues, experiences of hearing difficulties, and risk perceptions. They were also asked about their preferences in relation to typical venue sound levels and beliefs about other attendees’ preferences. Results showed that while participants generally rated their hearing as good, the majority had experienced hearing difficulties following sound exposure at music venues. The majority of regular patrons were dissatisfied with current sound levels, with around three-quarters of participants reporting preferences below the levels typically experienced at music venues. Participants were generally aware of the risk posed by high sound levels and those who regarded themselves to be at greater risk from attending music venues were more likely to prefer lower sound levels. These findings have important consequences for the development of hearing health initiatives within entertainment venues. Rather than motivating patrons to change their behavior, encouraging venues to meet their patrons’ needs and preferences may be a more successful strategy. Venue operators may find that this approach has a positive impact not only on the hearing health of patrons, but also on the economic health of their venue. Ultimately, reducing the hearing risk in music venues may best be achieved not by telling people what to do, but by listening to what they actually want.

## Introduction

Sound levels in live music venues and nightclubs have been consistently high since the 1960s and 1970s, when amplified guitars and monitor speakers appeared on stage at rock concerts, and stacks of multiple high-powered front-of-house speakers were used to convey the music to the audience. These technological changes resulted in sound levels of much higher intensity than had previously been possible. An early survey of sound levels in discotheques in the 1970s reported mean levels of 97 dB (Bickerdike and Gregory, 1980) and Clark’s (1991) review of several studies from the previous two decades showed that the mean of all reported sound levels from discotheques and rock concerts was 103.4 dBA (Clark, 1991). More recently, data from the National Acoustic Laboratories’ Non-Occupational Incidents Situations and Events (NOISE) database suggests that in Australian nightclubs, the continuous equivalent noise level (L_Aeq_) ranged from 82 to 106 dB L_Aeq_, mean: 96 dB L_Aeq_). For popular concerts, the range was 85–105 dB L_Aeq_, mean 95 dB L_Aeq_; and for live music in small venues (or “gigs”), the range was 86–102 dB L_Aeq_, with a mean of 94 dB L_Aeq_ (Beach et al., 2013).

These levels are similar to the intensity levels of power tools or heavy machinery, which produce sounds that are considered to be unpleasant and unwanted. Yet, loud music, despite being of similar intensity, is usually regarded more favorably, and managers of both live music venues and nightclubs produce loud music in the belief that their patrons find it desirable. Welch and Fremaux conducted interviews with regular nightclub attendees, DJs, musicians, sound engineers and bar managers, and found that loud music is desirable because it enhances positive emotions, masks unwanted negative emotions and helps people feel energized. It also facilitates socializing by removing inhibitions and promoting intimacy, especially in nightclub environments (Welch and Fremaux, 2017b). In some cases, loud music acts as a drug-like stimulant that is associated with addict-like responses in some individuals (Florentine et al., 1998; Schmuziger et al., 2012).

From the venue’s point of view, there may also be an economic rationale for playing music loudly. A number of studies have found that in loud music environments, people drink more quickly and consume more drinks than when music levels are moderate (McCarron and Tierney, 1989; Guéguen et al., 2004, 2008). The authors suggest that the high sound levels increase arousal, which in turn leads to an enhanced behavioral response, i.e., consuming more drinks more quickly. An alternative explanation is that patrons drink more quickly in high-noise environments because loud music makes conversation virtually impossible, and therefore drinking is the only viable option (Forsyth and Cloonan, 2008). Despite the potential benefits and attractions of playing loud music in entertainment venues, it also brings significant risks for patrons’ hearing. Typical levels are well above legislated workplace sound level limits (85 dB L_Aeq,8__h)_, and patrons often report tinnitus and temporary threshold shift (TTS) following these events. Although a one-off visit may not cause a long-term risk to hearing, animal studies (and some human studies) suggest that these volumes can result in synaptic damage between the cochlea and auditory nerve (Kujawa and Liberman, 2009; Liberman et al., 2016). For those who attend music venues on a regular basis, thereby exposing themselves to repeated and lengthy episodes of loud music, there is a risk of developing permanent hearing loss and/or tinnitus.

Various campaigns have attempted to inform patrons about the risk that these sound levels pose to hearing, e.g., Know your noise^1^, Dangerous decibels^2^, Don’t lose the music^3^. However, there is little evidence that these efforts have led to change in sound levels in venues, earplug use amongst patrons remains uncommon, and only a handful of jurisdictions have imposed an upper limit on amplified music levels (Tronstad and Gelderblom, 2016). Furthermore, anecdotal evidence suggests that patrons don’t seriously consider venue volumes in relation to risk because they harbor an expectation that levels are already monitored and/or restricted such that the threat to their hearing is minor. As a result, they may have relatively low motivation to limit their exposure from a risk perspective.

According to Welch and Fremaux (2017a), three parallel processes contribute to patrons’ acceptance of, or desire for, high sound levels in music venues. In the first place, there is adaptation, in which the auditory systems adapts to a loud sound environment such that it becomes easier to tolerate. Then conditioning occurs, in which there is a learned association between loud music, its benefits (arousal, social cohesion, masking of background noise, enabling intimacy) and the positive aspects of environments in which it is heard, thus people become conditioned to enjoy music at high volume levels. Finally, over time, a process of acculturation results in a pervasive expectation that loud music is an integral component of entertainment, and therefore music venues provide high volumes to meet consumer expectations.

Other social factors can also contribute to listening perceptions and reported preferences. Visiting music venues is considered a social activity for most attendees and participation is likely to take place with reference to perceived social norms of those considered to be fellow members of this “in-group.” According to social identity theory, individuals evaluate how well they fit within a group by categorizing the behaviors of that group (Hogg, 2000). Beliefs about group attitudes and actions subsequently influence the beliefs and behaviors of the individual. For patrons of music venues, the norms are likely to include the widely held presumption that patrons find high sound levels at nightclubs and live music venues desirable. Individual preferences will subsequently be influenced by an individual attendee’s beliefs about what they consider to be desirable to their group.

Importantly, an individual’s beliefs can be influenced by a social norm regardless of whether the perception of the norm is accurate. For example, previous research into preferred headphone listening levels suggests that sound level preferences can be influenced by *misperceptions* of social norms. Gilliver et al. (2012) showed that music listeners consistently (and incorrectly) believe that their peers listen at volumes higher than themselves. If a similar misperception occurs amongst music venue patrons, it may lead them to believe that others are more willing to accept and seek out higher volumes than they themselves desire, and that loud volumes are inevitable.

Regardless of the mechanisms and motivations that underlie the culture of playing music at high volumes, it remains unanswered whether patrons actually like music being played at levels typically found in venues. A recent British study of 325 students provided some indication that patrons would prefer lower sound levels, with 70.2% reporting that they believe sound levels in nightclubs should be limited to “safe volumes” (Johnson et al., 2014). A study conducted in Belgium showed that patrons preferred music played at 98 dB L_Aeq_ compared to 103 dB L_Aeq_ (Gilles et al., 2014) and an earlier study of Swiss young adults showed that between 43.3 and 52.2% of attendees believed that sound levels at discotheques, techno parties and music concerts were too high (Mercier and Hohmann, 2002). Previous work in our lab has shown that around two-thirds of patrons at a silent disco listening to music over headphones (where the sound level is not apparent to others) were satisfied with sound levels of 89–93 dB L_Aeq_ or lower (Sorensen et al., 2017). Such results are particularly significant when considering that even a small difference in volume can substantially reduce the associated risk. For example, sound levels of 101 and 95 dB L_Aeq_ may be perceived as similarly loud by patrons, but there is a four-fold increase in the level of risk when music is played at 101 dB compared to a 95-dB level.

In this study, regular attendees at nightclubs and live music venues were surveyed about typical and preferred sound levels to determine whether the sound levels are desired by the audience for whom they are intended. The aims of the study were to (a) determine whether attendees were satisfied with levels typically experienced at music venues, (b) ascertain their attitudes toward sound levels, (c) describe their current behaviors in relation to hearing protection, and (d) their willingness to adopt strategies to protect their hearing at music venues. A secondary aim was to assess whether the responses of nightclub attendees differed significantly from those of live music venue attendees.

## Materials and Methods

### Background

The data were collected in August 2012 from a large “citizen science” survey that was held during Australia’s National Science Week, an annual event that aims to promote science and technology throughout the community. The anonymous web-based survey was developed by the authors in conjunction with the Australian Broadcasting Commission (ABC)’s Science unit, who provided technical expertise and promotion of the survey.

The overall aim of the survey was to examine Australians’ hearing health, behaviors and beliefs relating to noise exposure, risk of hearing loss, and hearing health. The online survey, Sound Check Australia^4^, included several different topic areas or “modules” examining different aspects of hearing health. The survey was promoted throughout Australia, primarily through ABC media channels, and upon completion of the survey, respondents were eligible to enter a competition to win tickets to a music concert of their choice.

Prior to commencing the survey, visitors to the website were provided with information about the purpose of the study and the anonymous nature of the data collection procedure. Because the survey was conducted entirely online, it was not possible to obtain written consent, however all users were required to indicate that they had read the information prior to participating. Approval to conduct the study was obtained from the Australian Hearing Human Research Ethics Committee, in accordance with Australia’s *National Statement on Ethical Conduct in Human Research* (National Health and Medical Research Council [NHMRC], 2007).

### Participants

The survey was open to anyone aged 15 and over, and 9,904 people completed at least one module of the survey during the 5 weeks of the survey period. Respondents were aged 15–99 (mean age: 30.1, *SD* = 18.1, and just over half (50.3%) were male. Of the 9,904 respondents, 955 reported attending “a nightclub or dance club” or “a live gig or music performance at a smaller venue” at least twice per month, and they were subsequently invited to complete an additional module, which asked questions about their attendance at music venues. Those who regularly attended both types of venue (*n* = 174) were randomly assigned to answer questions related to either nightclubs or live music venues, but not both. Responses from 22 participants were excluded because the data were considered suspect, i.e., they contained inappropriate or spurious responses to one or more questions, resulting in a final dataset of 933 participants. Participants were drawn from all states and territories of Australia.

### Survey Items

In addition to reporting demographic information about age, gender, education, occupation, attendance at music venues, and self-reported hearing loss, this paper details responses from 15 survey items taken from the two modules that specifically focussed on either live music venues or nightclubs (see Tables 1, 2). Except where specified below, all survey items appeared in both modules, differing only in relation to whether they referred to “nightclubs” or “live music venues.” Items *a* and *b* asked about the symptoms of hearing damage experienced after attending the venues. Item *c* asked about the self-perceived risk associated with attendance at the venues, and item *d* asked respondents to select one of four options that best described their attitude toward typical sound levels at venues.

**Table 1:** Percentages of participant responses to items a–d and results of chi-square tests of independence.

	Response options	Nightclubs (%)	Live music venues (%)	*χ^2^*	*p*
***a.* TTS:** Have you ever noticed that you were not able to hear as well as usual, or that your ears felt ’blocked’ or ’dull’ following a visit to a nightclub/live music venue?	Never/almost never	9.7	8.2	5.18	0.27
	Occasionally	24.4	24.2		
	Sometimes	22.7	29.0		
	Frequently	28.2	25.5		
	Almost always	15.0	13.0		
	Total [at least occasionally]	90.3	91.8		
***b.* Tinnitus:** Have you ever experienced tinnitus (ringing in your ears) following a visit to a nightclub/live music venue?	Never/almost never	15.8	12.2	2.98	0.56
	Occasionally	21.6	22.3		
	Sometimes	23.1	24.7		
	Frequently	25.3	24.5		
	Almost always	14.2	16.2		
	Total [at least occasionally]	84.2	87.8		
***c.* Perception of risk:** Do you think the typical noise level at the nightclubs/live music venues you attend is harmful to your own hearing?	Unsure	1.4	0.8	0.91	0.82
	Not at all	2.7	2.7		
	A little	55.6	57.0		
	A lot	40.3	39.5		
***d.* Perception of sound levels:** Do you find that the music at most of the nightclubs/live music venues you go to is usually	Not as loud as you’d like	1.4	1.9	12.7	0.005^∗^
	Just right	12.8	18.3		
	Loud, but tolerable	59.7	62.6		
	Louder than you’d like	26.0	17.2		


*^∗^significant result.*

**Table 2:** Percentages of participants who “agree/strongly agree” with statements in items *e–l* and results of two-proportion *z*-tests.

	Nightclubs (%)	Live music venues (%)	*z*	*P*
***e.* Avoidance.** I avoid particular nightclubs/live music venues that I know play music too loud.	23.9	23.4	0.19	0.85
***f.* Attitude to loud music. NC:** When I go clubbing, I want to lose myself in the music. I’m there for a good time, I’m not thinking about my ears, my health, or anything else.	55.1	22.9	9.7	0.000^∗^
**LM:** I like my live music to be loud - the louder the better. I’m there for a good time, I’m not thinking about my ears, my health, or anything else.				
***g.* Ability to chat. NC:** When I go out, I want to chat with my friends as well as dance so I’d prefer it if there were some quieter places to sit and chat when we’re taking a break.	85.6	37.2	15.2	0.000^∗^
** LM:** When I go out to a live music venue, I want to chat with my friends as well as enjoy the music so I’d prefer it if the noise levels were lower.				
***h.* Free earplugs.** If nightclubs/live music venues were giving away free earplugs, I might wear them sometimes.	38.6	68.3	8.84	0.000^∗^
***i.* Warnings.** Some people say that nightclubs/live music venues should put up warning signs or show the sound level in decibels but I don’t see the point. It wouldn’t make any difference to my behavior.	46.0	42.0	1.19	0.23
***j.* Laws already in place.** I’m confident that existing laws and regulations are in place to ensure that nightclubs/live music venues only play music at safe levels.	25.2	21.3	1.37	0.17
***k.* Individual responsibility.** I don’t know what all the fuss is about. We all know nightclubs/live music venues are loud. It’s up to the individual to work out whether they want to spend time there or not.	50.7	53.5	0.82	0.41
***l.* Variation in sound levels.** If I had the choice, I’d like clubs to mix it up a bit, maybe play some really loud dance tunes, followed by a couple of more mellow tunes.	27.3	-	n/a	n/a


*^∗^significant.*

For items *e–l*, shown in Table 2, respondents used a 5-point scale from 1 (strongly disagree) to 5 (strongly agree) to indicate their level of agreement with various statements related to nightclubs and live music venues. Five items *(e, h, i, j, k)* were identical for both venues, but items *f* and *g* were worded differently to reflect the different venue types, and item *l* was presented for attendees of live music venues only.

**FIGURE 1:**
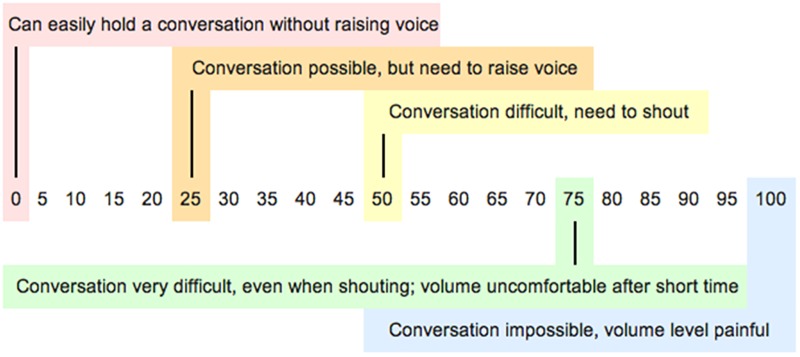
Scale describing different sound levels and how easy or difficult it would be to communicate with someone at arm’s length.

The final set of items (*m–o*) asked about typical and preferred sound levels at the venues. Respondents were presented with a subjective loudness rating scale (Figure 1) based on one validated previously (Beach et al., 2012; Williams et al., 2013), and found to provide a reasonable estimate of noise exposure when compared to objectively measured sound levels. Respondents were asked to answer the following questions: *m)* Provide a number between 0 and 100 to indicate the typical noise level at the live music venues you attend; *n)* Thinking of the other patrons at nightclubs/live music venues you attend, provide a number between 0 and 100 to indicate the noise level you think they would prefer; *o)* Imagine you were able to control the noise level at the nightclubs/live music venues you attend. Provide a number between 0 and 100 to indicate your preferred noise level at nightclubs/live music venues.

### Data Analysis

All statistical analyses were performed with Statistica version 13 (Dell, Tulsa, OK, United States). For survey items *a*–*d* and the question about self-reported hearing loss, chi-square tests of independence were used to determine whether there were significant differences in the responses of the two venue-type groups. For items *e*–*l*, two proportion *z*-tests were used to compare the percentages of participants who agreed with each statement across the two groups. For items *m*–*o, t*-tests were used to compare the typical and preferred sound levels of the two groups. Logistic regression was used to investigate the relationship between six predictor variables and the participants’ preference for lower sound levels in nightclubs and live music venues. The predictor variables were age, gender, degree of self-perceived hearing risk, self-rated hearing ability, and experience of TTS and tinnitus. To account for multiple comparisons and minimize Type I errors, the false discovery rate was set at 10% and the procedure described by Benjamini and Hochberg (1995) was followed.

## Results

### Nightclub Attendees

There were responses from 555 participants (42.6% female) with a mean age of 24.2 (15–69; *SD* = 8.1). Most participants lived in urban environments (85.8%), and 14.2% were located in rural areas. Participants were highly educated: 39% held a university degree; 17.3% held a trade qualification; and 34.7% had completed secondary school. More than a third (34.5%) were students and a similar proportion (34.5%) were professionals, managers or worked in sales; 27.9% worked in trade or clerical roles; and the remainder (3.1%) reported not working outside the home.

Respondents were frequent attendees of nightclubs, visiting on average 3.4 times per month (range: 2–28, *SD* = 2.6) and spending on average 4.6 h per visit. They also frequented popular concerts and live gigs more than once a month (concerts: average 1.3, *SD* = 1.5, live gigs 1.7, *SD* = 1.6).

### Live Music Venues Attendees

There were responses from 378 participants (43.8% female). The mean age of the sample was 29.1 (15–69; *SD* = 11.8). Most participants lived in cities and towns (86.6%), and 13.4% resided in rural areas. As a group participants were very highly educated: 50% held a university degree; 16.4% held a trade qualification; and 24.6% had completed secondary school. Just over a quarter (27.3%) were students and over a third (37.4%) were professionals, managers or worked in sales; 30.5% worked in trade or clerical roles; and the remainder (4.8%) were not working outside the home.

Respondents were frequent attendees of popular concerts and live music gigs. They attended popular concerts on average 1.3 times per month (range = 1–12, *SD* = 1.3) and live gigs on average 3.3 times per month on average (range: 2–30, *SD* = 2.6). Average time per visit for concerts was 3.8 h and for gigs 3.7 h. They were also frequent nightclub attendees visiting on average 2.2 times per month (*SD* = 2.1).

### Hearing Health

**FIGURE 2:**
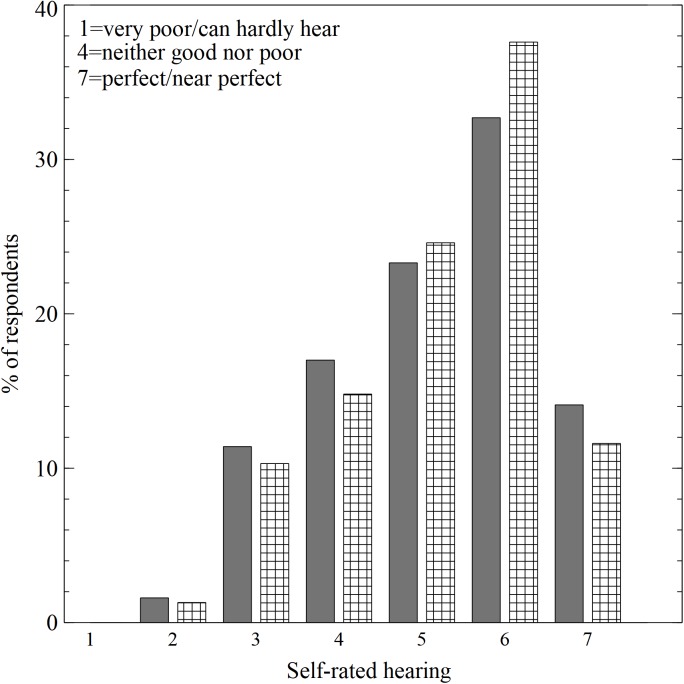
Distribution of self-rated hearing scores. Gray columns = nightclubs, cross-hatched columns = live music venues.

Participants were asked to describe their hearing ability using a 7-point rating scale, where 1 was “very poor/can hardly hear”; 4 was “neither good nor poor”; and 7 was “perfect/near perfect.” As shown in Figure 2, the majority reported that their hearing was good by selecting 5, 6 or 7, and there was no significant difference between the two groups ***χ^2^***= 3.69, *p* = 0.59.

As shown in Table 1, more than 90% of both nightclub and live music venue attendees had experienced TTS and more than 80% of both groups had experienced tinnitus after attending the respective venues. Chi-square tests of independence showed there was no significant difference between attendees of nightclubs versus attendees of live music venues in terms of respondents’ reported TTS and tinnitus.

### Perceived Risk

The perception of risk for both groups was nearly identical, with no significant differences between the groups. As shown in Table 1, the vast majority of respondents reported that they believed their hearing to be at risk, with around 40% of all participants reporting a high level of risk.

### Sound Levels

Table 1 shows the responses of participants when asked to select which of four options best described their attitude to typical sound levels in venues. The chi-square test showed a significant difference between the responses from the two groups (***χ^2^***= 12.7, *p* = 0.005). In particular, a smaller proportion of nightclub patrons thought that sound levels were appropriate compared to live music patrons. Around 1 in 4 nightclub patrons indicated that levels were “louder than you’d like them to be” whereas 1 in 6 live music venue attendees selected this option. In both groups, most respondents indicated that levels were “loud but tolerable.”

Table 2 shows the proportions of respondents who agreed with various statements relating to their attitudes and hearing health behaviors in nightclubs and live music venues. In most cases, there were no significant differences between the responses from each group. About a quarter of both groups had previously avoided noisy venues, and a similar proportion were confident that laws and regulations regarding safe sound levels were in place. Nearly half agreed that warning signs would not make a difference to their behavior and that individuals should take responsibility for their own hearing health. However, there were some issues on which the groups differed. Over half of nightclub attendees, but less than a quarter of live music venue attendees, agreed that they prioritized having a “good time” over their hearing health when attending entertainment venues (*z* = 9.7, *p* = 0.000). Nightclub attendees were also much more likely to agree that they would like a quiet place to chat, whereas less than 40% of live music patrons prioritized the need to talk to friends while at a venue (*z* = 15.2, *p* = 0.000). There was also a significant difference in the attitude held by the two groups in relation to free earplugs, with over two-thirds of live music attendees indicating an intention to wear earplugs compared to approximately one-third of nightclub attendees (*z* = 8.84, *p* = 0.000).

### Typical vs. Preferred Sound Levels

**FIGURE 3:**
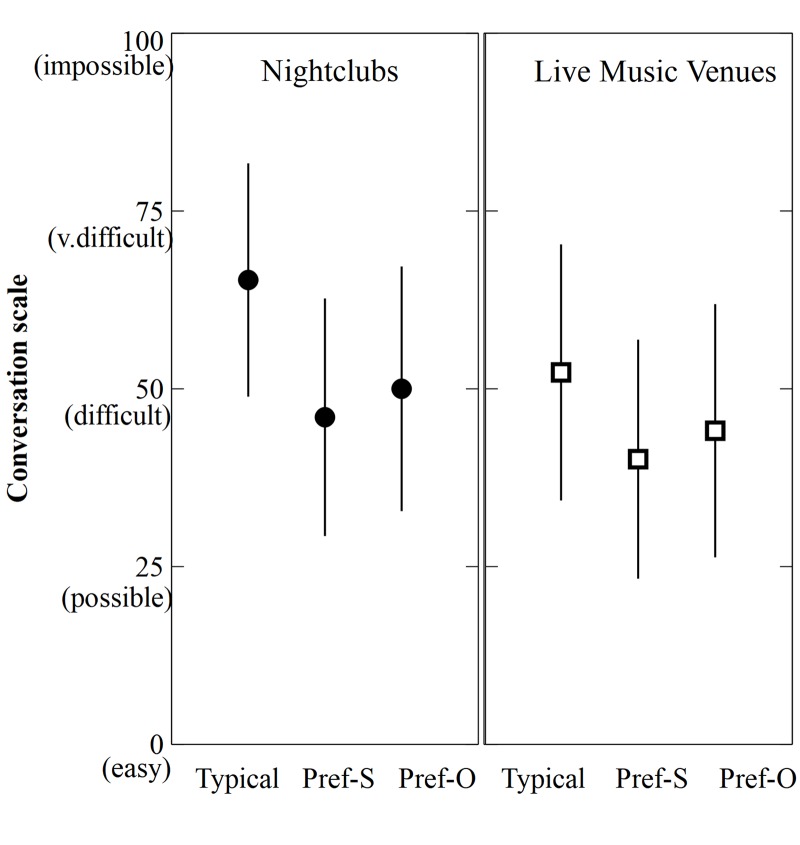
Participants’ mean estimated sound levels for live music venues and nightclubs. Typical = typical sound levels, Pref-S = preferred sound levels, Pref-O = Others’ preferred sound levels. Errors bars = +/-1 standard deviation.

For items *m*-*o*, participants were asked to indicate the typical and preferred sound level at nightclubs and live music venues they attend. A similar pattern of results was seen for both venues types, as shown in Figure 3. For nightclubs and live music venues, around three-quarters of all participants reported preferring a volume level that was significantly lower than their estimations of the typical sound level, and this difference was significant for both groups (nightclubs: *t*(548) = 19.98, *p* = 0.000; live music venues: *t*(373) = 13.74, *p* = 0.000). Both groups also reported believing that other attendees preferred a significantly higher sound level than their own preferred level (nightclubs: *t*(541) = 5.87, *p* = 0.000; live music venues: *t*(370) = 5.1, *p* = 0.000), although both were lower than the estimated typical levels.

Before conducting logistic regression, variance inflation figures (VIFs) were calculated to check for multicollinearity amongst the independent variables. All VIFs were between 1.02 and 4.25, which indicates that the level of multicollinearity was acceptable (Kutner et al., 2005). Logistic regression was performed to determine whether age, gender, perceived risk, experience of tinnitus and TTS, and self-reported hearing ability were related to participants’ preference for sound levels to be lower than typically found in nightclubs and live music venues. To calculate this, those who preferred a lower than typical volume were scored “1,” whereas those who preferred the same or higher volumes were scored “0.” As shown in Table 3, for both nightclubs and live music venues, self-perceived risk was a significant predictor. Those who considered themselves to be at no risk (or were unsure of the risk) were less likely to prefer lower sound levels than those who considered themselves to be at high risk (OR_nightclubs_ = 0.18, 95% CI: 0.07, 0.48; OR_livevenues_ = 0.03, 95% CI: 0.01, 0.14). Similarly, those who considered themselves to be at “a little” risk were less likely to prefer lower sound levels than those who rated themselves at high risk (OR_nightclubs_ = 0.57, 95% CI: 0.35, 0.93; OR_livevenues_ = 0.32, 95% CI: 0.18, 0.57). Male nightclub attendees were less likely to prefer lower sound levels compared to female attendees (OR = 0.54, 95% CI: 0.35, 0.85), and nightclub attendees who reported never, occasionally or sometimes experiencing tinnitus were less likely to prefer lower sound levels compared to those who experienced tinnitus more often (OR = 0.54, 95% CI: 0.31, 0.94). Live music attendees who reported never, occasionally, or sometimes experiencing TTS were also less likely to prefer lower sound levels compared to those who experienced TTS frequently or almost always, although this trend did not reach significance when the false discovery rate was applied.

**Table 3:** Odds ratios for six variables predicted to influence sound level preferences for nightclubs and live music venues.

	Nightclubs			Live Music		
**Predictor Variables**	**Odds ratio**	**95% CI**	***p***	**Odds ratio**	**95% CI**	***p***
**Age** (15-69)	1.01	0.98-1.04	0.39	1.00	0.98-1.02	0.96
**Gender** (male relative to female)	0.54	0.35-0.85	0.007^∗^	0.76	0.46-1.24	0.27
**Self-perceived risk**					
(“no risk/unsure” relative to “a lot”)	0.18	0.07-0.48	0.001^∗^	0.03	0.01-0.14	0.000^∗^
(“a little” relative to “a lot”)	0.57	0.35-0.93	0.03^∗^	0.32	0.18-0.57	0.000^∗^
**Self-rated hearing ability**					
(“poor” relative to “good”)	0.68	0.37-1.26	0.22	1.26	0.52-3.05	0.61
(“neither good nor poor” relative to “good”)	1.77	0.91-3.44	0.09	0.94	0.48-1.82	0.85
**TTS**					
(“never/occasionally/sometimes” relative to “frequently/almost always”)	0.72	0.43-1.21	0.22	0.55	0.30-1.01	0.05^ns^
**Tinnitus**					
(“never/occasionally/sometimes” relative to “frequently/almost always”)	0.54	0.31-0.94	0.03^∗^	1.57	0.87-2.85	0.14


*CI = confidence interval, ^∗^significant; ns = non-significant.*

## Discussion

The results presented here provide clear evidence that most regular patrons are dissatisfied with sound levels typically experienced in nightclubs and live music venues, consistent with earlier studies showing that many music venue attendees believe sound levels are “too high” (Mercier and Hohmann, 2002; Gilles et al., 2014; Johnson et al., 2014). Only 1 in 8 nightclub patrons and around 1 in 6 live music patrons indicated that they found typical levels were “just right,” with the vast majority of the remaining participants finding them either “tolerably loud” or “too loud.” This corresponds well with participants’ estimated sound levels, in which over 70% of participants preferred a sound level that was lower than the estimated typical level. Nightclub attendees generally reported higher volumes than live music venue attendees, with typical estimates centered around 65 – a level on the scale corresponding to considerable difficulty with conversation. For both venue types, there was a decline from “typical” to “preferred,” such that the mean preferred sound levels were between 40 and 45 – the point on the scale describing a volume at which conversation is possible. Although this study did not attempt to quantify the objective volume experienced by respondents at their respective venues, participants’ repeated subjective ratings using a simple conversational scale allowed for some important conclusions to be drawn in relation to sound level preferences. Our findings reflect that louder levels are generally experienced in nightclubs compared to live music venues, but more importantly, they show that while people expect to have difficulty talking in music venues, they would prefer volumes that fall well short of discomfort and pain.

Interestingly, for both venue types, the predicted preferred level for others was significantly higher than for themselves, suggesting that while the majority of patrons would like lower sound levels, many assume they are in the minority and that their preferred levels are lower than the norm. These results follow the predictions of social comparison theory, whereby perceived social norms can influence individuals’ behaviors. Attendee responses highlighted a belief on the part of individuals that the broader group of music venue attendees has a desire for higher volumes. This belief makes them less likely to ask for change and unwilling to make their wishes known, whether to venue operators or peers. This acceptance, in turn, can reinforce other participants’ perceptions of a group norm with a preference for high volumes.

Social comparisons can also create barriers to the adoption of protective behaviors. Individuals who perceive high volume exposure as an important aspect of group membership may be unwilling to risk their own membership by taking steps to reduce their noise exposure. Here we found that nightclub patrons were much less willing to consider using free earplugs than attendees of live music venues, even though both groups perceived themselves to be similarly at risk from their attendance. This suggests that there is a particularly strong social norm reinforcing earplug rejection amongst nightclubbers that prevails despite their awareness of the risks involved.

The loud music that is typical of many nightclubs provides an immersive experience for patrons, and unsurprisingly more than half agreed with the statement: “When I go clubbing, I want to lose myself in the music. I’m there for a good time, I’m not thinking about my ears, my health, or anything else.” In contrast, less than a quarter of live music patrons agreed with the equivalent statement: “I like my live music to be loud - the louder the better. I’m there for a good time, I’m not thinking about my ears, my health, or anything else.” This difference is likely reflective of the different motivations that patrons have for attending nightclubs vs live music venues, and the different social identities associated with each venue type. Live music patrons are often motivated by a desire to see a particular band or performer, and the focus is on the musical experience, whereas for nightclub patrons, they are more likely to be interested in dancing and socializing with friends. Because nightclubs are usually open well into the early hours of the morning, patrons often spend long periods of time in these venues, and it is therefore understandable that over 85% of attendees were in favor of quiet spaces where they could seek respite from the loud music and chat to friends. In contrast, live music patrons were less interested in having quiet opportunities to talk, either because they already have ample opportunities to talk in the quieter breaks between “sets” or perhaps because many would regard it as disrespectful to talk while a band or singer is performing.

Despite the differences in the sound levels in nightclubs and live music venues and the motivations of the different groups of clientele, the pattern of perceived risk was virtually identical in both groups. Only a very small fraction of participants believed that they were not at risk, with the majority acknowledging that attendance at music venues was a risky activity. Risk awareness is a crucial factor in promoting healthy behaviors of all kinds. One’s perceived susceptibility to a particular negative health outcome is a key element in most theories that attempt to explain decision-making with regard to health behaviors. Typically those who believe themselves to be at risk or susceptible to a negative outcome are the ones most likely to engage in protective health behaviors (Becker, 1974; Azjen and Fishbein, 1980; Janz and Becker, 1984). The same appears to hold true for hearing, with this study showing that those who believe themselves to be at greater risk are between twice and thirty times more likely to prefer lower sound levels than those with lower risk beliefs. This finding, and the fact that the experience of symptoms such as tinnitus and TTS were also associated with lower sound level preferences, suggests that effective hearing health messages are likely to be ones that incorporate the concepts of personalized risk and the experience of symptoms. In particular, we need to develop innovative and compelling messages that make risk tangible, and drive home the idea that temporary symptoms of damage can be pre-cursors to more permanent and irreversible consequences.

Of course, convincing patrons to change their behavior is only one approach to tackling the risk associated with high sound levels. Changing the behavior of venues is likely to be a more effective way of reducing risk for all patrons, regardless of their personal motivations. The results of this study point to a number of strategies which venues could adopt. Firstly, dropping the sound to a level where conversation is merely “difficult” rather than “impossible” in both nightclubs and live music venues is likely to meet with the approval of the majority of patrons. The drop in sound levels need not to be drastic. A drop of even 3 dB will halve the risk for all patrons, while satisfying their desire for lower sound levels. Another approach that venues could consider is the provision of free earplugs. Live music patrons in particular were supportive of this, with almost 7 in 10 indicating that they would consider using them if they were available, a finding consistent with recent research by Cha et al. (2015). Providing quiet rooms or “chillout” spaces, particularly in nightclubs, is another strategy to consider. Interestingly, providing warnings or decibel readings to inform patrons about sound levels received little support from the patrons surveyed here, with around 40% of both groups indicating that warnings would make little difference to them, although this is perhaps not surprising considering the relatively high level of risk awareness amongst the cohort.

These findings may also have implications for how best to design effective regulations to reduce the risk of hearing damage for music venue patrons. More than three-quarters of respondents expressed a lack of confidence in the ability of existing laws and regulations to ensure that venues play music at safe levels, and given that there are currently no sound level limits in place in Australian venues (apart from sound limits imposed under workplace and environmental law), their lack of confidence is well-founded. A number of European countries have moved toward regulating music venues and most of these require that venues limit their sound levels, and also provide free earplugs, quiet places, and warnings for patrons. If implemented in future, our results suggest that the success of any such measures will be largely dependent on the type of venue in which they are implemented. While some strategies are likely to be more successful in nightclubs (e.g., chillout rooms), other strategies (e.g., provision of earplugs) are likely to see greater uptake in live music venues. It is also worth noting that around half of the participants agreed that responsibility should rest with the individual, so measures which involve an element of patron choice or subtle improvements rather than a heavy-handed regulatory approach are likely to be met with less resistance.

### Limitations

When interpreting the results presented here, readers should take into account various limitations of the methods employed. Large-scale online surveys do have the potential to include data of a lower quality than that collected during face-to-face surveys. Although every effort was made to clear the data set of unreliable entries, some dubious responses may remain. The potential risk of including a small amount of unreliable data should be weighed against the benefits obtained from the relatively large sample from a broad population base that was only possible because the survey was administered online. Ideally, a test of patrons’ preferred sound levels (in dB L_Aeq_) while at music venues would enable a more direct correspondence between actual decibel levels and patrons’ sound level estimates that was not possible to obtain using the conversation-based sound level scale used here. Nevertheless, the levels reported by nightclub patrons compared to live music patrons are consistent with objectively measured levels, suggesting that the scale was well understood by respondents. Of course, the results are also limited by the degree to which the sample represents the wider population of music venue patrons in Australia. Restricting participation to those who reported regular recent attendance at music venues was an attempt to ensure that respondents were familiar with these venues, and not responding in relation to outdated memories and perceptions.

### Conclusion

This work clearly demonstrates that for both nightclubs and live music venues in Australia, typical sound levels are louder than desirable for the majority of patrons. Venues that listen to their patrons and drop their volumes will be satisfying the demands of a much wider audience, rather than catering to the wishes of a small minority who like it loud. Catering to this larger group of existing and potential new customers not only makes good economic sense, it will also help to shift the burden of responsibility for risk reduction from patrons. The results presented here suggest that a combination of patron-directed and venue-led initiatives are likely to be the most effective way of reducing risk from music venues. Lower volumes in all venues, chillout rooms in nightclubs and free earplugs in live music venues, coupled with innovative messages that focus on raising awareness of personal risk, while leveraging patrons’ widespread experience of TTS and tinnitus, should ultimately result in music venues that provide a satisfying musical experience without the risk of long-term hearing damage.

## Data Availability

The datasets generated for this study are available on request to the corresponding author.

## Author Contributions

EB and MG contributed to the study concept and design. EB drafted the manuscript and conducted the statistical analyses. Both authors revised the manuscript and approved the submitted version.

## Conflict of Interest Statement

The authors declare that the research was conducted in the absence of any commercial or financial relationships that could be construed as a potential conflict of interest.

## Footnotes

1 https://knowyournoise.nal.gov.au/

2 http://dangerousdecibels.org/

3 https://www.actiononhearingloss.org.uk/you-can-help/campaigns-and-influencing/campaigns-by-country/dont-lose-the-music/

4 https://www.nal.gov.au/soundcheck/

## References

[B1] Azjen I. Fishbein M. (1980). *Understanding Attitudes and Predicting Social Behavior.* Englewood Cliffs, NJ: Prentice-Hall.

[B2] Beach E. F. Gilliver M. Williams W. (2013). The noise (non-occupational incidents, situations and events) database: a new research tool. *Ann. Leisure Res.* 16 149–159. 10.1080/11745398.2013.793157

[B3] Beach E. F. Williams W. Gilliver M. (2012). The objective - subjective assessment of noise: young adults can estimate loudness of events and lifestyle noise. *Int. J. Audiol.* 51 444–449. 10.3109/14992027.2012.658971 22380619

[B4] Becker M. H. (1974). The health belief model and personal health behavior. *Health Educ. Monogr.* 2 324–508. 10.1177/109019817400200407

[B5] Benjamini Y. Hochberg Y. (1995). Controlling the false discovery rate: a practical and powerful approach to multiple testing. *J. R. Stat. Soc. Ser. B* 57 289–300. 10.1111/j.2517-6161.1995.tb02031.x

[B6] Bickerdike J. Gregory A. (1980). *An Evaluation of Hearing Damage Risk to Attenders at Discotheques.* Leeds: Polytechnical School of Constructional Studies.

[B7] Cha J. Smukler S. R. Chung Y. House R. Bogoch I. I. (2015). Increase in use of protective earplugs by rock and roll concert attendees when provided for free at concert venues. *Int. J. Audiol.* 54 984–986. 10.3109/14992027.2015.1080863 26609734

[B8] Clark W. W. (1991). Noise exposure from leisure activities: a review. *J. Acoust. Soc. Am.* 90 175–181. 10.1121/1.401285 1880286

[B9] Florentine M. Hunter W. Robinson M. Ballou M. Buus S. (1998). On the behavioral characteristics of loud-music listening. *Ear. Hear.* 19 420–428. 10.1097/00003446-199812000-00003 9867290

[B10] Forsyth A. Cloonan M. (2008). Alco-pop? The use of popular music in glasgow pubs. *Popular Music Soc.* 31 57–78. 10.1080/03007760601061902

[B11] Gilles A. Thuy I. De Rycke E. Van de Heyning P. (2014). A little bit less would be great: adolescents’ opinion towards music levels. *Noise Health* 16 285–291. 10.4103/1463-1741.140508 25209038

[B12] Gilliver M. Carter L. Macoun D. Rosen J. Williams W. (2012). Music to whose ears? The effect of social norms on young people’s risk perceptions of hearing damage resulting from their music listening behavior. *Noise Health* 14 47–51. 10.4103/1463-1741.95131 22517303

[B13] Guéguen N. Jacob C. Le Guellec H. Morineau T. Lourel M. (2008). Sound level of environmental music and drinking behavior: a field experiment with beer drinkers. *Alcohol. Clin. Exp. Res.* 32 1975–1978. 10.1111/j.1530-0277.2008.00764.x 18647281

[B14] Guéguen N. Le Guellec H. Jacob C. (2004). Sound level of background music and alcohol consumption: an empirical evaluation. *Percept. Mot. Skills* 99 34–38. 10.2466/pms.99.1.34-38 15446627

[B15] Hogg M. A. (2000). “Social identity and social comparison,” in *Handbook of Social Comparison: Theory and Research*, eds Suls J. Wheeler L. (Dordrecht: Kluwer Academic Publishers), 401–421. 10.1007/978-1-4615-4237-7_19

[B16] Janz N. K. Becker M. H. (1984). The health belief model: a decade later. *Health Educ. Q.* 11 1–47. 10.1177/109019818401100101 6392204

[B17] Johnson O. Andrew B. Walker D. Morgan S. Aldren A. (2014). British university students’ attitudes towards noise-induced hearing loss caused by nightclub attendance. *J. Laryngol. Otol.* 128 29–34. 10.1017/s0022215113003241 24398027

[B18] Kujawa S. G. Liberman M. C. (2009). Adding insult to injury: cochlear nerve degeneration after “temporary” noise-induced hearing loss. *J. Neurosci.* 29 14077–14085. 10.1523/jneurosci.2845-09.2009 19906956 PMC2812055

[B19] Kutner M. H. Nachtsheim C. J. Neter J. Li W. (2005). *Applied Linear Statistical Models.* Boston, MA: McGraw Hill.

[B20] Liberman M. C. Epstein M. J. Cleveland S. S. Wang H. Maison S. F. (2016). Toward a differential diagnosis of hidden hearing loss in humans. *PLoS One* 11:e0162726. 10.1371/journal.pone.0162726 27618300 PMC5019483

[B21] McCarron A. Tierney K. J. (1989). The effect of auditory stimulation on the consumption of soft drinks. *Appetite* 13 155–159. 10.1016/0195-6663(89)90112-8 2802594

[B22] Mercier V. Hohmann B. W. (2002). Is electronically amplified music too loud? What do young people think? *Noise Health* 4 47–55. 12537841

[B23] National Health and Medical Research Council [NHMRC] (2007). *National Statement on Ethical Conduct in Human Research 2007.* Canberra: National Health and Medical Research Council, Australian Research Council.

[B24] Schmuziger N. Patscheke J. Stieglitz R. Probst R. (2012). Is there addiction to loud music? Findings in a group of non-professional pop/rock musicians. *Audiol. Res.* 2:e11. 10.4081/audiores.2012.e11 26557326 PMC4630946

[B25] Sorensen R. Beach E. F. Gilliver M. Daugaard C. (eds) (2017). “Preferred listening levels: a silent disco study,” in *Proceedings of the International Symposium on Auditory and Audiological Research (ISAAR) 2017*, (Nyborg).

[B26] Tronstad T. Gelderblom F. (2016). Sound exposure during outdoor music festivals. *Noise Health* 18 220–228. 10.4103/1463-1741.189245 27569410 PMC5187664

[B27] Welch D. Fremaux G. (2017a). Understanding why people enjoy loud sound. *Semin. Hear.* 38 348–358. 10.1055/s-0037-1606328 29026266 PMC5634808

[B28] Welch D. Fremaux G. (2017b). Why do people like loud sound? A qualitative study. *Int. J. Environ. Res. Public Health* 14:908. 10.3390/ijerph14080908 28800097 PMC5580611

[B29] Williams W. Beach E. F. Gilliver M. (2013). Development of a subjective loudness rating scale. *Int. J. Audiol.* 52 650–653. 10.3109/14992027.2013.802382 23819616

